# Assessing the implementation of evidence-based alcohol policies on Atlantic Canadian post-secondary campuses: A comparative analysis

**DOI:** 10.17269/s41997-024-00907-4

**Published:** 2024-07-26

**Authors:** Kara Thompson, Stephanie Cooper, William Langille, Brynn Webber, Rachael MacDonald-Spracklin, Mark Asbridge, Bryce Barker, Darren Kruisselbrink, Janine Olthuis, Catherine Paradis, Sherry Stewart, Tim Stockwell, Robert Strang

**Affiliations:** 1 St. Francis Xavier University, Antigonish, NS Canada; 2 Dalhousie University, Halifax, NS Canada; 3 Canadian Centre on Substance Use and Addiction, Ottawa, ON Canada; 4 Acadia University, Wolfville, NS Canada; 5 University of New Brunswick, Fredericton, NB Canada; 6 Canadian Institute for Substance Use Research, Victoria, BC Canada; 7 Government of Nova Scotia, Halifax, NS Canada

**Keywords:** Alcohol policy, Post-secondary, Comparative analysis, Best practice, Politique sur l’alcool, postsecondaire, analyse comparative, pratique exemplaire

## Abstract

**Objective:**

This study assessed the quality of campus alcohol policies against best practice to assist campus decision-makers in strengthening their campus alcohol policies and reducing student alcohol use and harm.

**Methods:**

Drawing on empirical literature and expert opinion, we developed an evidence-based scoring rubric to assess the quality of campus alcohol policies across 10 alcohol policy domains. Campus alcohol policy data were collected from 12 Atlantic Canadian universities. All extracted data were verified by the institutions and then scored.

**Results:**

On average, post-secondary institutions are implementing only a third of the evidence-based alcohol policies captured by the 10 domains assessed. The average campus policy score was 33% (range 15‒49%). Of the 10 domains examined, only enforcement achieved an average score above 50%, followed closely by leadership and surveillance at 48%. The two heaviest-weighted domains—availability and access, and advertising and sponsorship—had average scores of 27% and 24%, respectively. However, if post-secondary campuses adopted the highest scoring policies from across all 12 campuses, they could achieve a score of 74%, indicating improvement is possible.

**Conclusion:**

Atlantic Canadian universities are collectively achieving less than half their potential to reduce student alcohol-related harm. However, this study identifies opportunities where policies can be enhanced or modified. The fact that most policies are present at one or more campuses highlights that policy recommendations are an achievable goal for campuses. Campuses are encouraged to look to each other as models for improving their own policies.

**Supplementary Information:**

The online version contains supplementary material available at 10.17269/s41997-024-00907-4.

## Résumé

**Objectif:**

L’étude a évalué la qualité des politiques relatives à l’alcool sur les campus par rapport aux pratiques exemplaires afin d’aider les décideurs des campus à renforcer leurs politiques relatives à l’alcool et à réduire la consommation d’alcool et les méfaits connexes dans la population étudiante.

**Méthode:**

En faisant appel à la littérature empirique et aux opinions d’experts, nous avons élaboré une grille de notation factuelle pour évaluer la qualité des politiques relatives à l’alcool sur les campus dans 10 domaines associés aux politiques sur l’alcool. Les données des politiques relatives à l’alcool sur les campus sont extraites des politiques de 12 universités du Canada atlantique. Toutes les données extraites ont été confirmées par les établissements, après quoi nous leur avons attribué une note.

**Résultats:**

En moyenne, les établissements postsecondaires ne mettent en œuvre que le tiers des politiques factuelles relatives à l’alcool faisant partie des 10 domaines évalués. La note moyenne des politiques des campus a été de 33 % (intervalle de 15 à 49 %). Des 10 domaines pris en compte, seule la mise en application a obtenu une note moyenne de plus de 50 %, suivie de près par le leadership et la surveillance, à 48 %. Les notes moyennes dans les deux domaines les plus lourdement pondérés (disponibilité et accès, et publicité et commandites) ont été de 27 % et de 24 %, respectivement. Toutefois, si les campus postsecondaires adoptaient les politiques les mieux notées des 12 campus, ils obtiendraient une note de 74 %; une amélioration est donc possible.

**Conclusion:**

Les universités du Canada atlantique réalisent collectivement moins de la moitié de leur potentiel de réduction des méfaits liés à l’alcool dans la population étudiante. Notre étude indique cependant des possibilités d’améliorer ou de modifier les politiques. Le fait que la plupart des politiques recommandées sont présentes sur un ou plusieurs campus montre qu’elles constituent un objectif réalisable. Nous encourageons chaque campus à améliorer ses propres politiques en s’inspirant de celles des autres.

## Introduction

Young adults consume alcohol more frequently and in higher quantities than at any other time in the lifespan, particularly post-secondary students (Carter et al., 2010; Hingson et al., 2016). Biological predisposition to risk-taking behaviour during young adulthood, along with environmental (e.g., reaching the legal drinking age) and social determinants, such as living independently for the first time and the desire to socialize with peers, contributes to heavy alcohol use among post-secondary students (Krieger et al., 2018). According to the 2019/2020 Canadian Postsecondary Education Alcohol and Drug Use Survey (CPADS), 60% of Canadian post-secondary students had engaged in heavy drinking in the past 30 days (Health Canada, 2019) and 56% of students reported experiencing at least one alcohol-related harm in the past month (Health Canada, 2019).

Students’ heavy alcohol use is associated with increased risk of unintentional (e.g., falls, traffic collisions) and intentional (e.g., interpersonal violence, self-harm) injuries, risky sexual behaviour (e.g., unprotected sex, multiple partners), sexual assault, visits to the emergency department for alcohol-related toxicity, and death (Brown et al., 2016; King et al., 2022; White & Hingson, 2013). The negative effects of heavy alcohol use also extend beyond the drinker. Between 30% and 70% of post-secondary students reported experiencing harm from other students’ alcohol use, such as being threatened or assaulted or having their sleep or studies interrupted (Davis-MacNevin et al., 2017; Health Canada, 2019; Thompson et al., 2017). Student binge drinking is also associated with substantial economic costs, to the institution and the community, such as property damage and costs for additional resources such as security and disciplinary services (Perkins, 2002).

Alcohol policies that serve the interests of public health and social well-being are an essential population-based prevention strategy and one of the most effective alcohol harm countermeasures at the national level (Babor et al., 2010). The evidence is clear that when alcohol is inexpensive (e.g., happy hours) and easily accessible (e.g., high density of outlets), and when alcohol policies are poorly enforced (e.g., age verification/ID’ing), rates of alcohol use and related harms are high in the general population (Hahn et al., 2010; MD Collaborative, 2013; Nelson et al., 2005). Policies with the greatest evidence of effectiveness are those that reduce both economic and physical availability of alcohol (Anderson et al., 2009; McMillan et al., 2016; Naimi et al., 2014). Studies on campus-specific alcohol policies are more limited, but research suggests that effective population-based policies can successfully be applied to the campus environment (Hahn et al., 2010; MD Collaborative, 2013).

Campus alcohol policies help shape student alcohol use and the drinking culture on campus, and post-secondary institutions have a responsibility to provide safeguards for students that support their well-being and protect them from harm (Henderson et al., 2019). However, there is substantial variability in alcohol policies and how they are enacted and enforced across campuses (Dejong & Langford, 2002; Nova Scotia Department of Health and Wellness, 2012). Furthermore, campus policies are often implemented without knowledge and/or evidence of their effectiveness (Jernigan et al., 2019). Jernigan and colleagues (2019) investigated the accessibility, clarity, and effectiveness of alcohol policies at 15 United States colleges. Findings showed that less than half of the campus policies being implemented were rated as “effective” (defined as policies likely to comprehensively affect the physical and/or normative drinking environment on campus as rated by a Delphi panel of alcohol experts), and alcohol policies tended to be hard to access, spread across multiple webpages, and written in high reading-level language and jargon that would be difficult to understand without a college degree.

Previous assessments of campus alcohol policies have largely been limited to assessing the presence or absence of specific alcohol policies (Faden et al., 2009; Hirschfeld et al., 2005; Mitchell et al., 2005). Jernigan et al. (2019) is the only study to date to consider policy effectiveness in their assessment of campus alcohol policies; however, evidence of effectiveness was based solely on expert opinion. Past studies have also been limited to US institutions, which have distinct alcohol policy contexts relative to Canada, such as a legal drinking age of 21 (instead of 18/19 in Canada) and a much more prevalent and influential Greek system (e.g., fraternities and sororities) (Jernigan et al., 2019; MD Collaborative, 2020). To address these limitations, the current study applies a well-established comparative approach used by the Canadian Alcohol Policy Evaluation (CAPE) project to assess provincial and territorial alcohol policies (Giesbrecht et al., 2013; Naimi et al., 2023; Stockwell et al., 2019). This approach draws upon existing empirical evidence to develop a detailed scoring rubric to assess the quality of current alcohol policy implementation compared to best practice across several alcohol policy domains. Now in its third iteration, CAPE provides detailed reports outlining jurisdictional (Canadian provinces and territories, federal government) policy strengths and areas of improvement to assist decision-makers in implementing effective alcohol policies (Naimi et al., 2023). These reports have led to significant policy discussions and efforts towards policy improvement (Vallance et al., 2022). This comparative model has similarly been applied by MADD Canada to assess impaired driving policies, and by the Canadian Harm Reduction Policy Project to assess variation in and quality of Canadian harm reduction policies (Hyshka et al., 2017; MADD Canada, 2015; Wild et al., 2017).

The objective of the current study, the Campus Alcohol Policy Project (CAPP), is to provide comprehensive and evidence-based alcohol policy recommendations that campus decision-makers can use to strengthen their existing policies and reduce heavy drinking and related harms among their students. To do this, we developed an evidence-based scoring rubric to assess the quality of campus alcohol policies across 10 domains and employed this scoring rubric to rate campus alcohol policy effectiveness and comprehensiveness across 12 Atlantic Canadian universities.

## Methods

### Development of the scoring rubric

The research team was comprised of nine established alcohol and alcohol policy experts from post-secondary institutions in Atlantic Canada, as well as collaborators from the Canadian Centre on Substance Use and Addiction (CCSA), the Canadian Institute for Substance Use Research, and a Chief Medical Officer of Health in Nova Scotia. The 10 alcohol policy domains were informed by the WHO Global Strategy to Reduce the Harmful Use of Alcohol, the CAPE project, and the Postsecondary Education Partnership Alcohol Harms (PEP-AH) framework (Barker, 2017; Naimi et al., 2023; WHO, 2010). Each domain is outlined in Table 1, alongside the corresponding domains from the three guiding projects or frameworks. Each research team member was assigned a domain topic and tasked with conducting a narrative literature review to identify the most up-to-date evidence-based alcohol harm reduction policies and practices (Sukhera, 2022). Study inclusion was limited to the last 10 years at the time of searching, and the search engines PubMed, Web of Science, MEDLINE, and PsycINFO were used. Using the narrative synthesis of evidence, the research team developed a set of evidence-based policy and practice indicators for each domain. See Supplementary Table 1 for policies considered best practice. Each domain was scored out of 10. The scoring rubric underwent external peer review by a Research Methods Specialist from the Centre for Addiction and Mental Health and one of the primary methodologists on the CAPE project, Ashley Wettlaufer.

**Table 1:** Outline of the scoring rubric’s domains and corresponding domains from CAPE, WHO and PEP-AH

CAPP domain	CAPP definition	Domains from informing frameworks/projects		
		CAPE	WHO	PEP-AH
Availability & Access	Policies which are intended to reduce the convenience of alcohol, such as regulating outlet density, hours and days of sale, delivery of alcohol and where alcohol is permitted to be consumed (Cronce at el., 2018; Duthie et al., 2023; WHO, 2010)	Physical Availability	Availability of Alcohol	Availability & Marketing
Advertising & Sponsorship	Policies to reduce the volume and quantity of advertising, restrict the content of ads, and regulate industry sponsorship (Naimi et al., 2023; Paradis et al., 2020)	Marketing & Advertising Controls	Marketing of Alcoholic Beverages	
Harm Reduction	Policies aimed at reducing the negative consequences associated with alcohol use such as banning drinking games, implementation of safe-ride programs, and providing alternative alcohol-free programming (Fell et al., 2020; Jernigan et al., 2019; Layland et al., 2019; Monahan et al., 2019)	N/A	Reducing the Negative Consequences of Drinking and Alcohol Intoxication	Health Promotion, Prevention & Education
Pricing	Policies intended to reduce the availability of cheap alcohol, including setting a minimum unit price per standard drink, and prohibiting price promotions (Baldwin et al., 2014; Sharma et al., 2017; Thompson et al., 2017)	Pricing & Taxation	Pricing Policies	Pricing of Alcohol
Campus Services	Policies to ensure the availability of evidence-based alcohol prevention and intervention services across the continuum of care, from health promotion to treatment and recovery (MD Collaborative, 2013; Staton et al., 2018)	Intervention & Referral	Health Services Response	Campus Services
Bar & Events Practices	Policies intended to create safe and responsible drinking environments and include staff training, safe service policies, and clear procedures for events (Fell et al., 2017; Nova Scotia Department of Health and Wellness, 2012)	Liquor Law Enforcement	N/A	N/A
Community Action	Policies to strengthen cooperation and collaboration between campuses and the communities in which they reside (Martin et al., 2012; Saltz et al., 2010)	N/A	Community Action	Community Action
Leadership & Surveillance	Policies intended to ensure that campus alcohol policies are accessible and up to date, and assist in prioritizing alcohol policy initiatives on campus (Jernigan et al., 2019; Naimi et al., 2023)	Monitoring & Reporting; Alcohol Strategy	Monitoring & Surveillance; Leadership, Awareness & Commitment	N/A
Health & Safety Messages	Policies to ensure students are provided or have access to critical information to make informed choices about their alcohol consumption (Lopez et al., 2022; MD Collaborative, 2020)	Health & Safety Messaging	Leadership, Awareness & Commitment	Health Promotion, Prevention & Education
Enforcement	Policies to outline procedures for identifying and handling policy violations (Harris et al., 2010; Jones-Webb et al., 2014; Toomey et al., 2011)	Liquor Law Enforcement	Drink-driving Policies & Countermeasures	N/A

*CAPP* Campus Alcohol Policy Project, *CAPE* Canadian Alcohol Policy Evaluation, *WHO* World Health Organization, *PEP-AH* Postsecondary Education Partnership — Alcohol Harms

### Determining the weight of policy domains

The 10 policy domains were weighted based on (1) the strength of the evidence for a policy domain’s potential to reduce alcohol-related harm (“Effectiveness”) and (2) the proportion of the population that was expected to be affected by a particular policy if it was fully implemented (“Reach”). Following CAPE methodology (Naimi et al., 2023), the Delphi technique was used to determine appropriate domain weights. Team members independently and anonymously rated the degree of effectiveness and reach of each domain on a 5-point Likert scale with higher scores indicating greater effectiveness and greater reach, respectively. Team members discussed their ratings to reach consensus. Weights were calculated by multiplying the ratings for effectiveness and reach (possible range 0–25) and were applied to total scores (Supplementary Table 2).

### Data collection

Research assistants conducted online searches to collect publicly available campus alcohol policy documents from all 12 anglophone post-secondary institutions in the four Atlantic provinces (Nova Scotia, Prince Edward Island, New Brunswick, and Newfoundland and Labrador) in May 2022. Student enrollment at the 12 universities ranged from approximately 800 students to 13,000 students. Policy information was extracted and then sent to campus partners to verify the accuracy and completeness of the data, and/or provide missing data.

### Scoring data

Using the scoring rubric, data were scored independently by two members of the research team. Discrepancies between raters were discussed and resolved. In instances where no documentation or data was available for a specific indicator, it was assumed that no policy was in place and a score of zero was applied. Indicator scores were summed to yield domain scores out of 10. The final score (out of 100) for each campus was the sum of each domain score weighted for effectiveness and reach and is expressed as a percentage.^1^ Additionally, the highest scoring domains across all campuses were combined to calculate the “highest possible policy score” as an indicator of feasibility. This score represented the highest possible score attainable if all the best policies currently being implemented by at least one campus were adopted at a single institution.

## Results

### Total scores

**Fig. 1:**
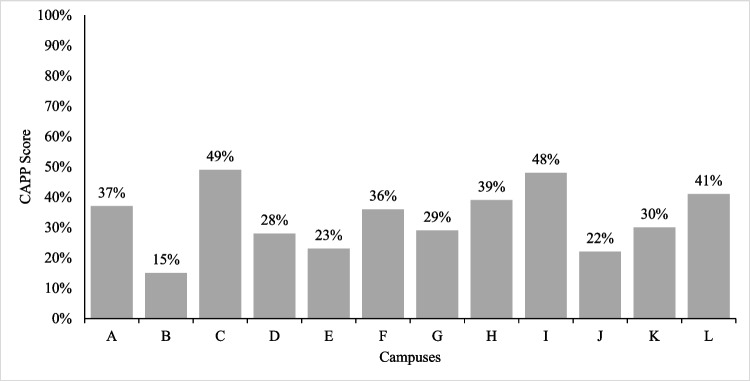
Total alcohol policy score by campus. Note: Each letter represents 1 of 12 Atlantic Canadian universities. Names were removed to preserve anonymity

On average, the 12 Atlantic Canadian universities were implementing only a third of the evidence-based alcohol policies captured by the 10 domains assessed. The average campus policy score was 33% and all campuses scored below 50% (range, 15‒49%). Figure 1 presents the total campus alcohol policy score for each institution.

### Domain scores

**Fig. 2:**
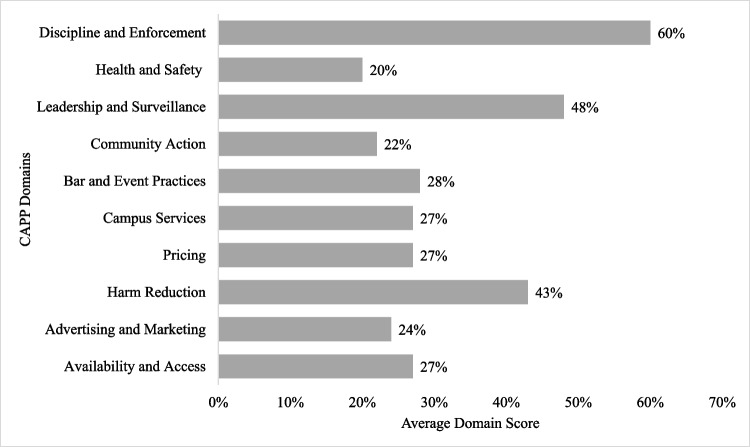
Average campus score by domain

Figure 2 reports the average scores for each of the 10 policy domains. The Enforcement domain had the highest score (60%) and was the only domain score that exceeded 50%. This was followed by Leadership & Surveillance, which scored the second-highest average (48%) and Harm Reduction, which scored the third-highest average (43%). All other domains scored below 30%, with the three lowest-scoring domains being Advertising and Sponsorship (24%), Community Action (22%), and Health & Safety Messages (20%). Key findings from each of the 10 domains are detailed below. Recommendations for strengthening campus alcohol policies in each domain can be found in Supplementary Table 3.

#### Availability and access

Scores in this domain ranged from 15% to 45% (*x̅* = 27%). Eleven campuses had an on-campus bar and, in keeping with best practice, 10 of these campuses were within the recommended density limits of one licensed establishment per 10,000 students. However, no campus policies specified density limit or the hours and days of sale, and 10 campuses had hours that extended before 4 pm and/or beyond 1 am. All 11 campuses allowed for on-campus delivery of alcohol.

#### Advertising and sponsorship

Domain scores ranged from 0% to 50% (*x̅* = 24%). Campuses generally scored poorly in all domain indicators. At most campuses (75%), advertising policies did not extend to all advertising mediums (e.g., print, digital, broadcast) or all vendors (e.g., on-campus bars, off-campus bars, alcohol manufacturers). Eight of twelve campuses (67%) did not have a policy regulating sponsorship.

#### Harm reduction

Scores on this domain varied from 10% to 80% (*x̅* = 42.5%). Consistent with best practice, nine campuses (75%) had a safe-ride program and eight offered bystander training (intervention training to teach individuals how to recognize and safely intervene in situations where someone is at risk of sexual violence (Fleming & Wiersma-Mosley, 2015)) for students (67%). In terms of harm minimization policies and practices, 10 campuses did not allow drinking games, six hosted dry orientation week, and six prohibited kegs of beer or other alcoholic drinks to be purchased and consumed on campus. However, only three campuses had formalized alcohol-free programming and events available for students.

#### Pricing

Scores ranged from 0% to 60% (*x̅* = 27%). Prices at campus bars generally exceeded provincial minimum unit prices and aligned with best-practice recommendations of $3.76 per standard drink. For spirits, all eight campuses with price data had a price per standard drink between $5.33 and $8.29; *x̅* = $6.93). For domestic beer products, six (of eight) campuses had a price per standard drink and prices ranged from $3.40 to $5.50 (*x̅* = $4.74). However, all campuses permitted happy hour discounts.

#### Campus services

Scores ranged from 0% to 80% (*x̅* = 27%). In keeping with best practice, eight campuses provided counselling services and/or referral options for substance use treatment and six campuses had a student-led medical response team trained in first aid. However, only one campus subscribed to an evidence-based prevention and intervention program (i.e., eCHECKUP to go) and no campus had a campus recovery community (Staton et al., 2018).

#### Bar and events

Scores ranged from 0% to 60% (*x̅* = 28%). In general, policies in this domain did not align with best practice. Only four of the eleven campuses with licensed establishments had a policy that required their staff to have responsible beverage training and fewer than half of campuses had alcohol control policies or safe service policies. For events, eight campuses had clear policies and procedures for alcohol use at on-campus events, but only half of these had procedures for off-campus events. Six campuses had a designated risk assessment committee or individual responsible for event approval.

#### Community action

Scores ranged from 0% to 60% (*x̅* = 21.7%). Almost all campuses (75%) were affiliated members of PEP-AH, led by the CCSA. PEP-AH was intended to share best practices and strategies to reduce alcohol-related harm on campuses; however, PEP-AH has not been active since the pandemic and currently has no funding. Only one of 12 campuses had an established campus-community coalition (8%), and three campuses collaborate with local law enforcement (25%), while no campuses partner with emergency services (0%).

#### Leadership and surveillance

Scores ranged from 0% to 70% (*x̅* = 48). Consistent with best practice, 10 campuses had a stand-alone alcohol policy (83%), and eight had an explicit harm reduction mandate. Six campuses also regularly updated their policy by a formal committee every 3 to 5 years. However, all policies received a “very difficult to read” score on the Flesch reading ease test (Brewer, 2019). In terms of monitoring and surveillance, nine campuses collected data on student substance use and harms regularly; however, no campus reported on the data findings.

#### Health and safety messages

Scores ranged from 10% to 40% (*x̅* = 20%). In keeping with best practice, seven campuses had a designated webpage for alcohol education with resources for students seeking help with alcohol and tips to minimize harm. However, the quality of these websites varied considerably. Few campuses mandated alcohol education for students. Four campuses had mandatory alcohol education for residence assistants and three had mandatory alcohol education for incoming students. No campus required health and safety messages to be displayed at on-campus bars or on alcohol containers sold in bars.

#### Enforcement

Scores ranged from 45% to 75% (*x̅* = 60%). All campuses had procedures for handling policy infractions and designated personnel to determine sanctions, and 11 campuses had clear and specific sanctions for policy violations. In most cases, sanctions became more severe for repeat or multiple infractions (eight campuses). However, eight campuses did not have procedures for identifying or reporting policy infractions.

### Highest possible policy score

As an indicator of feasibility, the highest possible policy score was calculated as the highest score achievable if the highest scoring policies from across all 12 campuses were adopted by a single institution. The high possible policy score was 74%, indicating improvement is possible (Table 2).

**Table 2:** Highest policy score by domain

Policy domain	Highest policy score %
Availability and access	70
Advertising and sponsorship	60
Harm reduction	95
Pricing	60
Campus services	80
Bar and event practices	70
Community action	80
Leadership and surveillance	80
Health and safety	40
Enforcement	100
Total score	74%

## Discussion

This study was the first comprehensive assessment of the quality of campus alcohol policies in Canada. Modelled after CAPE (Giesbrecht et al., 2013; Naimi et al., 2023), campus alcohol policies were gathered and scored against internationally established best practices. Findings suggest that on average, evidence-based alcohol policies are not being widely adopted across Atlantic Canadian campuses. Campuses are reaching less than half their potential to reduce student alcohol use and related harms. Consistent with findings from US colleges (Jernigan et al., 2019), campus policies were difficult to find, distributed piecemeal across multiple documents, and outdated. The inaccessibility of campus alcohol policies made it difficult for researchers to gather information and, more importantly, likely impedes students’ ability to stay informed.

Of the 10 domains examined, only enforcement achieved an average score above 50%, followed closely by leadership and surveillance at 48%. Unfortunately, these two highest scoring domains were also the lowest weighted in terms of effectiveness and reach. The two heaviest-weighted domains—availability and access, and advertising and sponsorship—had average scores under 30%. These findings align with CAPE’s evaluation of provincial and territorial policies, showing that decision-makers tend to adopt fewer effective policies, and overlook the most effective, because the former tend to be easier to implement and more supported by consumers (Giesbrecht et al., 2013; Naimi et al., 2023; Stockwell et al., 2019). Campus decision-makers should work to prioritize the implementation of policies within the most effective domains.

A more encouraging result is that most of the alcohol policies assessed across the 10 domains were being successfully implemented on one or more campuses. For example, one campus scored 10/10 on leadership and surveillance and another scored 9.5/10 on harm reduction. If post-secondary campuses learned from one another and adopted the existing best-practice alcohol policies from other campuses, they could all achieve a more favourable overall score of 74%. This highest policy score highlights that the policies evaluated as part of this project are feasible and present an achievable goal for campuses. Campuses should look to each other as models for improving their own policies. The revival of PEP-AH at the national level would help create opportunities for campuses to learn from each other and assist campuses in strengthening their collaborations.

The overall goal of this project was to assess the quality of campus alcohol policies against best practice to help campuses strengthen their policies and reduce student alcohol use and harm. To achieve this, each campus was provided a personalized campus-specific report and a virtual webinar which summarized their findings (including policy strengths and weaknesses), ranked them in relation to the other participating campuses, and outlined our specific recommendations for policy change. Beyond these campus-specific recommendations, there are various overall recommendations we are making for all participating campuses (Supplementary Table 3). Some of the biggest policy gaps that remain across campuses include a lack of policies that control student exposure to alcohol advertising, and policies that ensure that students have access to alcohol education and health and safety messages. Alcohol marketing directly contributes to the perpetuation of alcogenic cultures by glamourizing and promoting alcohol consumption, while these cultures in turn often downplay or ignore the risks associated with excessive alcohol use (Hydes et al., 2020; Petticrew et al., 2017). As such, these policy areas are essential to implement for shifting the current alcogenic culture on campuses. Campuses also need to invest in evidence-based upstream approaches to prevent the development of problematic alcohol use among students, and establish campus supports for students in recovery from substance use (campus recovery communities). Finally, campuses should ensure that their bar staff are properly trained in safe service practices and non-violent crisis intervention. These policy areas should be priorities for all campuses involved in the current study.

### Limitations

This study has several limitations. Narrative reviews were conducted to identify evidence-based best practice and policy and as such, this study lacked systematic methods for gathering and synthesizing this evidence. Moreover, while the scoring rubric went through an external peer-review process, feedback and recommendations were only obtained from one peer reviewer. Third, all data included in this study were verified by campus stakeholders in May 2022. However, policies may have changed since that time. Further, it was assumed that if a policy could not be found on the website, it did not exist. However, there were instances where campuses indicated a policy existed, but documentation could not be provided or found. The decision to assign zeros in this case might have led to an underestimation of campus scores. However, given that undocumented alcohol policies are unlikely to be adhered to or sustained over time, our scores highlight the urgent need for campuses to document current alcohol policies and practices. It is also important to acknowledge that scores do not reflect the degree to which a policy is implemented in practice or enforced. For example, a campus may ban alcohol advertising on campus, but may not closely monitor for compliance or penalize individuals or groups in violation. Moreover, some policy measures may or may not be contextually appropriate depending on the campus. For example, some universities do not have an on-campus licensed establishment; hence, certain policy measures did not pertain to those institutions. In these instances, indicators considered not applicable were excluded when calculating scores, and the domain scores and total scores were prorated accordingly.

## Conclusion

While federal and provincial alcohol policies are some of the most effective tools for shaping the drinking culture of all citizens, there remain considerable gaps in various alcohol policy areas (Naimi et al., 2023). Post-secondary institutions play a significant role in shaping the alcohol culture on campus and are uniquely positioned to go beyond provincial and jurisdictional policies to fill policy gaps (Krupa et al., 2019). Unfortunately, when we look at what could be done and compare it with what is actually being done, we have to conclude that campuses are collectively achieving less than half their potential to reduce student alcohol use. This analysis provides campuses with tailored recommendations that can be used to strengthen their campus alcohol policies in support of student health. The fact that much of what is being recommended has already been implemented in at least one campus underscores the feasibility of improvement. To achieve the best results, campuses should take at least some action in each of the 10 policy domains. Campuses are also encouraged to conduct regular assessments of their alcohol policies and carefully document policy changes to evaluate their impact on student drinking. Future research aims to develop a digital self-assessment tool to allow other institutions to conduct a self-evaluation of their alcohol policies.

## Contributions to knowledge

What does this study add to existing knowledge?

- This study was the first to comprehensively assess the quality of alcohol policies within Canadian post-secondary education institutions.
- All 12 Atlantic Canadian universities received a failing grade, achieving less than half their potential to reduce alcohol-related harm. Policy scores were further undermined by the fact that policies were difficult to find, distributed piecemeal across multiple documents, and outdated.
- Most of the policies in this assessment were being implemented by at least one institution, indicating that positive change is feasible and attainable. Campuses are encouraged to look to each other as models for improving their own policies.

What are the key implications for public health interventions, practice, or policy?

- This study underscores the necessity for Atlantic Canadian universities to adopt comprehensive alcohol control measures to reduce and prevent alcohol-related harms among students.
- Findings highlight examples of best practice and identified opportunities where campus alcohol policies can be enhanced or modified.
- Comprehensiveness alcohol policies should include indicators from all 10 policy domains to optimize effectiveness and reach.

## Supplementary Information

Below is the link to the electronic supplementary material.

Supplementary file1 (DOCX 47 KB)

## Data Availability

Available upon request.

## Footnotes

1 In instances where an indicator was not applicable to the institution, the score was prorated.

## Code availability

Available upon request.
