# Supplementary material for: Assessing the implementation of evidence-based alcohol policies on Atlantic Canadian post-secondary campuses: A comparative analysis
Source: Can J Public Health. 2024 Jul 26;115(5):789–800. doi: 10.17269/s41997-024-00907-4 (PMC11535009; doi:10.17269/s41997-024-00907-4)
Supplement: Supplementary file 1 — Supplementary file1 (DOCX 47 KB) [file 41997_2024_907_MOESM1_ESM.docx]

**Supplementary Table 1**

*Scoring rubric policy and practice indicators and associated scoring for each policy domain*

| **Domain** | **Best Practice** | **Score** |
| --- | --- | --- |
| Availability & Access [1-7] | Policy restricts the number of licensed establishments on campus. | 1 |
|  | One outlet or fewer per 10,000 students. | 2 |
|  | Open ≤5 days per week. Hours of operation limited to 4pm - 1am. | 3 |
|  | Alcohol is prohibited in public places, sporting events, student recruitment events; Substance-free residence options available. | 2 |
|  | Delivery of alcohol is prohibited. | 1 |
|  | Student card cannot be used to purchase alcohol. | 1 |
| Advertising & Sponsorship [1, 8-10] | Ban on all forms of advertising | 2 |
|  | Restrictions apply to all vendors (campus bars, off-campus bars, student groups, alcohol manufacturers) | 1 |
|  | Regulations include restrictions on ad placement, advertisement of price, contests, and pictures/text of alcohol. | 2 |
|  | Ban on all sponsorship from the alcohol industry | 2 |
|  | Procedure for monitoring compliance with advertising regulations. | 2 |
|  | Students are prohibited from holding positions as brand ambassadors | 1 |
| Harm Reduction [4, 10-18] | No pub crawls; no drinking games; dry orientation week; no BYO events; no kegs; Friday morning classes | 3 |
|  | Provide and support the implementation of alcohol-free programming | 2 |
|  | Bystander training required and implemented widely on campus. | 1 |
|  | Campuses who have a stated amnesty policy. | 1 |
|  | Explicit mandate to conduct campus patrols during high-risk times. | 1 |
|  | Campuses have a safe ride program for students to get home safely. | 1 |
|  | Campuses have an organized student-led harm reduction initiative. | 1 |
| Pricing [8, 19-24] | Average minimum pricing per standard drink on campus is ≥$3.76 | 3 |
|  | Drink specials, discounts, and price promotions are prohibited. | 2 |
|  | Lower strength products are priced less than higher strength products. | 2 |
|  | Gifting of alcohol is prohibited. | 1 |
|  | Campus bars provide free or discounted non-alcoholic products | 2 |
| Campus Services [3, 10, 25] | Campuses subscribe to an evidence-based universal prevention program (AlcoholEdu; Alcohol-Wise, eCHECKUP to go; Alcohol 101) | 2 |
|  | Campuses subscribe to an evidence-based targeted prevention program for at-risk students (BASICS; Alcohol skills training; eCHECKUP to go; SBIR) | 2 |
|  | Specialized alcohol use treatment available on campus or by referral. | 2 |
|  | Campuses have a student medical response team trained in first aid that attends high-risk events and responds to on-campus emergencies. | 2 |
|  | Campus Recovery Community for students in recovery. | 2 |
| Bar & Events Practices [8, 10, 25-30] | Mandatory beverage service and security training for all staff | 1 |
|  | Training recertification required | 1 |
|  | ≥4 of the following: government ID required; no admittance after 12am; refuse/remove intoxicated patrons; special procedures for high-risk events; ensure intoxicated patrons get home safe | 2 |
|  | All 9 safe service policies: limits on number of drinks; no bulk purchases; limit drink container size; food is available; no last call; no shots; 1oz mixed drinks; lower alcohol products available. | 3 |
|  | Shared oversight of campus bars by the student union and institution | 1 |
|  | Clear procedures for on-campus events and off-campus events; risk assessment committee; mandatory security at events that serve alcohol | 2 |
| Community Action [1, 31-34] | Established campus and community coalition to address alcohol-related local issues. | 4 |
|  | Campus security has an established relationship with law enforcement. | 2 |
|  | Affiliated with alcohol-related organizations with a specific mandate to minimize or reduce alcohol related harm on campuses. | 2 |
|  | Partnership with local health services to enhance on campus safety | 2 |
| Leadership & Surveillance [1, 4, 10, 18, 35, 36] | Campus has a stand-alone alcohol policy | 1 |
|  | Alcohol policy can be found online in less than 30 seconds | 2 |
|  | Policy scored 60-70 on Flesch Reading Ease Test | 2 |
|  | Policy has an explicit harm reduction/safe consumption mandate | 1 |
|  | Alcohol policy is regularly updated by a committee every 3-5 years. | 1 |
|  | Identified leader for monitoring alcohol harm and policy implementation | 1 |
|  | Annually collect data on student alcohol use and harms | 2 |
| Health & Safety Messages [25, 37-43] | Alcohol containers have health information labels | 2 |
|  | Display of health and safety messages mandatory on campus | 2 |
|  | Designated alcohol education page on campus website | 2 |
|  | Alcohol education covers the risks of acute intoxication, drinking moderately, social norms, drinking and driving risks, pregnancy risks, harm to others, available health services | 2 |
|  | Alcohol education is mandatory for incoming students, residence assistants, athletics, event host and volunteers. | 2 |
| Enforcement [4, 44-48] | Procedures/programs for identifying and reporting policy infractions | 2 |
|  | Designated personnel and procedures for handling policy infractions | 1 |
|  | There are sanctions for individual and organizational violations. Sanctions are tied to specific policies, increase in severity with repeat violations, and extend to off-campus policy violations | 5 |
|  | Clear communication of local/provincial alcohol policies. | 2 |

**Supplementary Table 2**

*Domain weights*

| Policy Domain | Effectiveness  (Out of 5) | Reach  (Out of 5) | Maximum Possible Score  (Out of 25) |
| --- | --- | --- | --- |
| 1. Availability & Access | 4 | 4 | 16 |
| 2. Advertising & Sponsorship | 3.5 | 4.5 | 15.75 |
| 3. Harm Reduction | 3 | 3.5 | 10.5 |
| 4. Pricing | 4.5 | 2 | 9 |
| 5. Campus Services | 3 | 3 | 9 |
| 6. Bar & Events Practices | 2.5 | 3.5 | 8.75 |
| 7. Community Action | 2.5 | 3.5 | 8.75 |
| 8. Leadership & Surveillance | 2.5 | 3.5 | 8.75 |
| 9. Health & Safety Messages | 2 | 4 | 8 |
| 10. Enforcement | 2.5 | 2 | 5 |
| **Overall Maximum Possible Score** | | | **99.5** |

*Maximum possible score (MPS) = Effectiveness * Reach

**Supplementary Table 3**

*Policy recommendations by domain*

| **Policy Domains** | **Recommendations** |
| --- | --- |
| Availability & Access | Close campus bars one to two days per week |
|  | Offer substance-free residence options |
|  | Prohibit the use of student cards to purchase alcohol. |
|  | Open campus bars a maximum of 7 hours per day with limited availability early in the day (before 4pm) and late at night (>1 am) to avoid opening hours coinciding with course offerings. |
|  | Prohibit the sale and consumption of alcohol at sporting events. Ban delivery of alcohol on campus. |
| Advertising & Sponsorship | Ensure advertising policies apply to all mediums, including digital platforms and promotional items. |
|  | Prohibit off-campus vendors from advertising on campus. |
|  | Develop clear procedures for monitoring compliance with campus & provincial advertising regulations (i.e., pre-approval process compliance checks). |
|  | Restrict the location of ad placement to licensed areas and prohibit the advertisement of price. |
|  | Clearly specify the terms of alcohol sponsorship to minimize youth exposure to branded content, particularly in sporting venues. |
|  | Ban all alcohol advertising and sponsorship on campus |
| Harm Reduction | Prohibit pub crawls. |
|  | Introduce a Good Samaritan policy. |
|  | Conduct regular campus patrols at high-risk times. |
|  | Establish and support a student-led harm reduction initiative on campus. |
|  | Fund and staff a formalized program that provides fun and safe alcohol-free experiences for students. |
| Pricing | Alcoholic products are priced at the recommended minimum unit price of $3.76 per standard drink. |
|  | Ban gifting of free alcohol |
|  | Price lower-alcohol products less than higher-alcohol products. |
|  | Ban price discounts (e.g., “happy hour”) at campus bars and events. Offer 'happy hour' discounts for food and non-alcoholic beverages instead |
| Campus Services | Subscribe to an evidence-based prevention program and develop a clear plan for dissemination. |
|  | Establish a student-led medical response team. |
|  | Create a collegiate recovery program. |
| Bar & Event Practices | Create a policy to ensure all staff serving alcohol receive responsible beverage service training and security measure training. |
|  | Require recertification of training biennially. |
|  | Implement the follow control policies: Patrons required to show government issued ID; No “admittance after 12am”, special operating procedures for high-risk events; refuse entry of intoxicated patrons; ensure intoxicated patrons get home safe. |
|  | Expand safe service policies to include setting limits on number of drinks, prohibiting last call, and ensuring lower strength alcohol products are available. |
|  | Develop clear policies and procedures for off-campus events. |
|  | Ensure institutional oversite of on-campus licensed establishments. |
|  | Prohibit the sale of pitchers and shots. |
| Community Action | Establish a campus-community coalition that involves community stakeholders such a local law enforcement and health care providers. |
| Leadership & Surveillance | Create a single comprehensive alcohol policy |
|  | Update your campus alcohol policy every 3 – 5 years. |
|  | Ensure the campus alcohol policy is easy to find on the institutional website and easy to read. |
|  | Regularly collect student alcohol use data by participating in the CPADS or NCHA surveys. |
|  | Report and utilize data about student alcohol use and harms to inform policy change. |
| Health & Safety Messages | Implement mandatory alcohol education for residence assistants, incoming students, athletes, and hosts of events serving alcohol. |
|  | Mandate displaying alcohol health and safety messages in on-campus licensed establishments, in residence, and at events serving alcohol. |
|  | Develop a webpage dedicated to alcohol education that provides in-depth evidence-based information on alcohol use and associated risks, as well as how to minimize these risks. |
|  | Add health information labels to alcohol containers sold on campus |
| Enforcement | Create specific procedures to monitor compliance with campus alcohol policies. For example, spot checks at events by campus security. |
|  | Create a complaint system for individuals to report suspected policy violations. |
|  | Develop sanctions for organizations and event hosts who violate campus alcohol policies. |
|  | Hold students accountable for policy violations that occur off-campus within the community. |

**References**

1. World Health Organization. Strategies to reduce the harmful use of alcohol: draft global strategy. [Internet]. Geneva: World Health Organization, 2010 March 25 [cited 2024 Apr 17]. Available from https://www.drugsandalcohol.ie/14097/1/WHO_strategies_to_re duce_alcohol_harm.pdf
2. Kypri K, Maclennan B, Connor J. Alcohol Harms over a Period of Alcohol Policy Reform: Surveys of New Zealand College Residents in 2004 and 2014. Int J Environ Res Public Health. 2020 Feb;17(3):836.
3. Cronce JM, Toomey TL, Lenk K, Nelson TF, Kilmer JR, Larimer ME. NIAAA’s College Alcohol Intervention Matrix. Alcohol Res. 2018 Jan;39(1):43–7.
4. Jernigan DH, Shields K, Mitchell M, Arria AM. Assessing Campus Alcohol Policies: Measuring Accessibility, Clarity, and Effectiveness. Alcohol Clin Exp Res. 2019 May;43(5):1007–15.
5. Bormann CA, Stone MH. The effects of eliminating alcohol in a college stadium: the Folsom Field beer ban. J Am Coll Health. 2001 Sep;50(2):81–8.
6. Barry AE, Russell A, Howell S, Phan P, Reyes D, Bopp T. (Unintended) Consequences of initiating an alcohol sales policy at college football stadiums: A case study. J Am Coll Health. 2019 Jul;67(5):397–401.
7. Colbert S, Wilkinson C, Thornton L, Feng X, Richmond R. Online alcohol sales and home delivery: An international policy review and systematic literature review. Health Policy. 2021 Sep;125(9):1222–37.
8. Stockwell, T., Wettlaufer, A., Vallance, K., Chow, C., Giesbrecht, N., April, N., Asbridge, M., Callaghan, R.C., Cukier, S., Davis-MacNevin, P., Dube, M., Hynes, G., Mann, R., Solomon, R., Thomas, G., Thompson, K. (2019). Strategies to Reduce Alcohol-Related Harms and Costs in Canada: A Review of Provincial and Territorial Policies. Victoria, BC: Canadian Institute for Substance Use Research, University of Victoria.
9. Kypri K, Maclennan B, Connor J. Alcohol Harms over a Period of Alcohol Policy Reform: Surveys of New Zealand College Residents in 2004 and 2014. Int J Environ Res Public Health. 2020 Feb;17(3):836.
10. Nova Scotia Department of Health and Wellness. Reducing Alcohol Harms Among University Students: A summary of Best Practices. [Internet]. Halifax: Government of Nova Scotia, 2012 [cited 2024 Apr 17]. Available from: https://novascotia.ca/dhw/addict ons/documents/Reducing-alcohol-harms-among-university- students.pdf
11. Patrick ME, Maggs JL, Osgood DW. LateNight Penn State Alcohol-Free Programming: Students Drink Less on Days They Participate. Prev Sci. 2010 Jun;11(2):155–62.
12. Wilkinson LL, Talbott LL. Late-Night Alcohol-Free Programming and Implications for Alcohol Prevention Among College Students: An Applications of the MCMOS Model. College Student Journal. 2018 Sep 22;52(3):384–97.
13. Read JP. What’s in a game? Future directions for the assessment and treatment of drinking games. Am J Drug Alcohol Abuse. 2014 Sep;40(5):415–8.
14. Ward RM, Cleveland MJ, Messman-Moore TL. Latent Class Analysis of college women’s Thursday drinking. Addict Behav. 2013 Jan;38(1):1407–13.
15. Ward RM, Bonar RN, Taylor EA, Witmer KA, Brinkman CS, Cleveland MJ, et al. Thursday drinking and academic load among college women. J Stud Alcohol Drugs. 2013 Nov;74(6):941–9.
16. Morean ME, Darling N, Smit J, DeFeis J, Wergeles M, Kurzer- Yashin D, et al. Preventing and Responding to Sexual Misconduct: Preliminary Efficacy of a Peer-Led Bystander Training Program for Preventing Sexual Misconduct and Reducing Heavy Drinking Among Collegiate Athletes. J Interpers Violence. 2021 Apr;36(7–8):NP3453–79.
17. Haas AL, Wickham RE, McKenna K, Morimoto E, Brown LM. Evaluating the Effectiveness of a Medical Amnesty Policy Change on College Students’ Alcohol Consumption, Physiological Consequences, and Helping Behaviors. J Stud Alcohol Drugs. 2018 Jul;79(4):523–31.
18. Fell JC, Scolese J, Achoki T, Burks C, Goldberg A, DeJong W. The effectiveness of alternative transportation programs in reducing impaired driving: A literature review and synthesis. J Safety Res. 2020 Dec;75:128–39.
19. Thompson K, Stockwell T, Wettlaufer A, Giesbrecht N, Thomas G. Minimum alcohol pricing policies in practice: A critical examination of implementation in Canada. J Public Health Policy. 2017 Feb;38(1):39–57.
20. Sharma A, Sinha K, Vandenberg B. Pricing as a means of controlling alcohol consumption. Br Med Bull. 2017 Sep 1;123(1):149–58.
21. Martinetti MP, Caughron RL, Berman HL, André J, Sokolowski MBC, Wiley S, et al. The Behavioral Economics of Alcohol Demand in French and American University Students. Alcohol Clin Exp Res. 2019 Mar;43(3):531–44.
22. Baldwin JM, Stogner JM, Miller BL. It’s five o’clock somewhere: An examination of the association between happy hour drinking and negative consequences. Subst Abuse Treat Prev Policy. 2014 Apr 23;9:17.
23. Blackwell AKM, De-Loyde K, Hollands GJ, Morris RW, Brocklebank LA, Maynard OM, et al. The impact on selection of non-alcoholic vs alcoholic drink availability: an online experiment. BMC Public Health. 2020 May 6;20(1):526.
24. Canadian Centre on Substance Abuse (CCSA). What Students Think about Drinking and Alcohol Policies: Feedback on the #RethinkTheDrink Talkback Tour. [Internet]. Ottawa: Canadian Centre on Substance Abuse, 2019 April [cited 2024 Apr 17]. Available from: https://www.ccsa.ca/sites/default/files/2019-04/CCSA-Students- Drinking-Alcohol-Policies-Summary-Report-2017-en.pdf
25. Maryland Collaborative to Reduce College Drinking and Related Problems. Reducing alcohol use and related problems among college students: A guide to best practices. 3rd ed [Internet]. Maryland: Maryland Department of Health, 2020 [cited 2024 Apr 17]. Available at: https://marylandcollaborative.org/project/guide-best- practices/
26. Quigg Z, Bigland C, Ross-Housel K, Hughes K, Bellis MA. STOP- SV: a training programme to prevent nightlife-related sexual violence (Evaluation Report). [Internet]. Liverpool: Public Health Institute, Liverpool John Moores University, 2018 Nov [cited 2024 Apr 17]. Available from: http://www.irefrea.eu/uploads/STOP- SV/Output-4.5_%20Finalreport-on-training-evaluation.pdf
27. Powers RA, Leili J. Bar Training for Active Bystanders: Evaluation of a Community-Based Bystander Intervention Program. Violence Against Women. 2018 Oct;24(13):1614–34.
    50. Chinman M, Ebener P, Burkhart Q, Osilla KC, Imm P, Paddock SM, et al. Evaluating the impact of Getting to Outcomes-Underage Drinking on prevention capacity and alcohol merchant attitudes and selling behaviors. Prev Sci. 2014 Aug;15(4):485–96.
28. Fell JC, Fisher DA, Yao J, McKnight AS. Evaluation of a responsible beverage service and enforcement program: Effects on bar patron intoxication and potential impaired driving by young adults. Traffic Inj Prev. 2017 Aug 18;18(6):557–65.
29. Haggård U, Trolldal B, Kvillemo P, Guldbrandsson K. Implementation of a multicomponent Responsible Beverage Service programme in Sweden-a qualitative study of promoting and hindering factors. Nordic Studies on Alcohol and Drugs. 2015 Feb 1;32(1):73–90.
30. Gillam SW. Nonviolent crisis intervention training and the incidence of violent events in a large hospital emergency department: an observational quality improvement study. Adv Emerg Nurs J. 2014;36(2):177–88.
31. Wolfson M, Champion H, McCoy TP, Rhodes SD, Ip EH, Blocker JN, et al. Impact of a Randomized Campus/Community Trial to Prevent High-Risk Drinking among College Students. Alcohol Clin Exp Res. 2012 Oct;36(10):1767–78.
32. Saltz RF, Paschall MJ, McGaffigan RP, Nygaard PMO. Alcohol Risk Management in College Settings: the safer California universities randomized trial. Am J Prev Med. 2010 Dec;39(6):491–99.
33. Flewelling RL, Grube JW, Paschall MJ, Biglan A, Kraft A, Black C, et al. Reducing Youth Access to Alcohol: Findings from a Community-Based Randomized Trial. Am J Community Psychol. 2013 Mar;51(1-2):264–77.
34. Martin BA, Sparks M, Wagoner K, Sutfin EL, Egan K, Sparks A, Rhodes SD, O’Brien MC, Easterling D, Wolfson M. Study to Prevent Alcohol-Related Consequences: Using a community organizing approach to implement environmental strategies in and around the college campus: An intervention manual. [Internet]. Winston-Salem: Wake Forest School of Medicine, 2013 [cited 2024 Apr 17].
35. Canadian Centre on Substance Abuse. Reducing the harms related to alcohol on Canadian campuses. [Internet]. Ottawa, ON: Canadian Centre on Substance Abuse, 2016 [cited 2024 Apr 17]. Available from: https://www.ccsa.ca/sites/default/files/2019- 05/CCSAPostsecondary- Education-Partnership-Alcohol-Harms-Strategy-2016-en.pdf
36. Faulkner G, Ramanathan S, Kwan M, Arasaratnam G, Bottorff J, Burnett A, et al. Developing a coordinated Canadian post-secondary surveillance system: A Delphi survey to identify measurement priorities for the Canadian Campus Wellbeing Survey (CCWS). BMC Public Health. 2019 Jul 11;19(1):935.
37. Scott JL, Brown AC, Phair JK, Westland JN, Schüz B. Self- affirmation, intentions and alcohol consumption in students: a randomized exploratory trial. Alcohol Alcohol. 2013;48(4):458– 463.
38. Strohman AS, Braje SE, Alhassoon OM, Shuttleworth S, Van Slyke J, Gandy S. Randomized controlled trial of computerized alcohol intervention for college students: role of class level. Am J Drug Alcohol Abuse. 2016;42(1):15–24.
39. Lopez SV, Leffingwell TR, Dunn DS, Warner EA. Intentions to Reduce Alcohol Use Following Brief Alcohol-Related Health Messages Among College Students. J Stud Alcohol Drugs. 2022 Nov;83(6):944–8.
40. Morgenstern M, Dumbili EW, Hansen J, Hanewinkel R. Effects of alcohol warning labels on alcohol-related cognitions among German adolescents: A factorial experiment. Addict Behav. 2021 Jun;117:106868.
41. Coomber K, Hayley A, Giorgi C, Miller PG. A Qualitative Investigation of Australian Young Adult Responses to Pictorial and Graphic Alcohol Product Warnings. Journal of Drug Issues. 2017 Oct 1;47(4):622–37.
42. World Health Organization. The SAFER technical package: five areas of intervention at national and subnational levels. Geneva: World Health Organization, 2019 [cited 2024 Apr 17]. Available from: https://iris.who.int/bitstream/handle/10665/330053/978924151 6419- eng.pdf?sequence=1
43. López-Olmedo N, Muciño-Sandoval K, Canto-Osorio F, Vargas-Flores A, Quiroz-Reyes A, Sabines A, et al. Warning labels on alcoholic beverage containers: a pilot randomized experiment among young adults in Mexico. BMC Public Health. 2023 Jun 15;23(1):1156.
44. Jones-Webb R, Nelson T, McKee P, Toomey T. An implementation model to increase the effectiveness of alcohol control policies. Am J Health Promot. 2014;28(5):328–35.
45. Carey KB, Carey MP, Maisto SA, Henson JM. Computer Versus In-Person Intervention for Students Violating Campus Alcohol Policy. J Consult Clin Psychol. 2009 Feb;77(1):74–87.
46. Harris SK, Sherritt L, Van Hook S, Wechsler H, Knight JR. Alcohol policy enforcement and changes in student drinking rates in a statewide public college system: a follow-up study. Subst Abuse Treat Prev Policy. 2010 Aug 4;5:18.
47. Knight JR, Harris SK, Sherritt L, Kelley K, Van Hook S, Wechsler H. Heavy Drinking and Alcohol Policy Enforcement in a Statewide Public College System. Journal of Studies on Alcohol. 2003;64(5):696–703.
48. Toomey TL, Miazga MJ, Lenk KM, Erickson DJ, Winters KC, Nelson TF. Enforcing alcohol policies on college campuses: reports from college enforcement officials. J Drug Educ. 2011;41(3):327–44.
